# Selected Mechanisms of Action of Bacteriophages in Bacterial Infections in Animals

**DOI:** 10.3390/v17010101

**Published:** 2025-01-14

**Authors:** Renata Urban-Chmiel, Ewelina Pyzik

**Affiliations:** Department of Veterinary Prevention and Avian Diseases, Faculty of Veterinary Medicine, University of Life Sciences in Lublin, 20-033 Lublin, Poland; renata.urban@up.lublin.pl

**Keywords:** bacteriophages, anti-phage antibodies, bacteria, infections

## Abstract

Bacteriophages, as ubiquitous bacterial viruses in various natural ecosystems, play an important role in maintaining the homeostasis of the natural microbiota. For many years, bacteriophages were not believed to act on eukaryotic cells; however, recent studies have confirmed their ability to affect eukaryotic cells and interact with the host immune system. Due to their complex protein structure, phages can also directly or indirectly modulate immune processes, including innate immunity, by modulating phagocytosis and cytokine reactions, as well as acquired immunity, by producing antibodies and activating effector cells. They can therefore have a profound impact on the course of bacterial infections by stimulating and at the same time inhibiting the systemic pro-inflammatory response. This review article presents a characterization of the processes by which bacteriophages affect selected immune mechanisms in selected animal species. The results of our own experiments using calves are also presented as examples. The paper contains many new examples of potential uses of bacteriophages and their effects on eukaryotic cells, especially in the course of bacterial infections, which are extremely important in experimental treatments exploiting phages as alternatives to antibiotics. The positive results of the effects of bacteriophages on eukaryotic cells during infections open up promising new prospects for their use as natural tools in the treatment of bacterial, fungal, and viral diseases in animals and humans.

## 1. Introduction

Bacteriophages are also known as bacterial viruses, and their life cycles are inextricably linked to the bacterial cells serving as their hosts. Due to the specific nature of their functioning, resulting from their lack of specific enzymatic structures, bacteriophages cannot exist independently outside the bacterial host cell. Phages are ubiquitous in various natural ecosystems, and their presence has also been confirmed in the bodies of animals and humans, e.g., in the mouth, the gastrointestinal tract, and the respiratory system, as well as in urine and serum. Their roles include maintenance of the homeostasis of natural ecosystems [1].

Bacteriophages were first described as separate particles at the beginning of the 20th century, at about the same time, by two independent scientists, Félix d’Hérelle [2] and Frederick William Twort [3]. At that time, phages were recognized as being effective in treating bacterial infections. However, due to Alexander Fleming’s discovery of penicillin in 1928 and the subsequent development of the antibiotic era, work on phage therapy was abandoned for many years, especially in Western countries. However, research on the development of bacteriophages continued to be conducted in Eastern European countries, for instance, at the Eliava Institute of Bacteriophages, Microbiology, and Virology (EIBMV) of the Georgian Academy of Sciences, in Tbilisi, Georgia, and the Hirszfeld Institute of Immunology and Experimental Therapy (HIIET) of the Polish Academy of Sciences, in Wroclaw, Poland [4,5].

Currently, due to the crisis induced by the globally increasing occurrence of drug resistance in bacteria, which poses a threat to humans and animals, bacteriophages may become one of the key alternatives to antibiotics, with practical potential in controlling bacterial infections [6]. Moreover, bacteriophages are the most abundant viral components in the bodies of humans and animals [7,8] and can play a key role in maintaining homeostasis in eukaryotic organisms [9].

According to the International Committee on Taxonomy of Viruses [10], the class Caudoviricetes currently comprises four identified orders of bacterial viruses, *Crassvirales*, *Kirjokansivirales*, *Methanobavirales*, and *Thumleimavirales*, as well as one order described as ‘unidentified’. Within the four identified orders, 14 families, 11 subfamilies, 59 genera, and 96 species have been identified. In the order classified as ‘unidentified’, 33 families have been distinguished, as well as an additional ‘unidentified’ family, 48 subfamilies, 690 genera, and 2156 species [11].

Experimental phage treatments in humans and animals show varied therapeutic efficacy and are very safe in the treatment of numerous diseases caused by pathogenic bacteria, from the treatment of burn wounds and infections of the skin and subcutaneous tissue to urinary tract, lung, and gastrointestinal infections [12].

### 1.1. Phages and Eukaryotic Cell Interactions

For many years, the prevailing opinion was that bacteriophages did not act on eukaryotic cells and exhibited tropisms only for bacteria serving as hosts for a given phage. Recent research, however, confirms completely new traits of bacteriophages, including the potential to penetrate mucous membranes and interact with the immune system of humans and animals [13]. For example, bacteriophage T4 has developed mechanisms of action on human mucosal immunity [14]. The study cited showed that this mechanism is possible due to binding of the phage to mucin, which restricts the ability of pathogenic bacteria to colonize epithelial cells and increases the phage’s capacity for subdiffusive motion on the mucosal surface and penetration into specific areas of the mucus, making it more effective in killing bacteria [15].

The effects of phages on the cells of complex organisms include their effects on immune tolerance, mucosal immunity, and homeostasis of the intestinal microbiome [16].

Apart from the extracellular antibacterial effects of bacteriophages, there have also been studies confirming that phages can inactivate intracellular bacteria. For example, a study conducted by Kaur et al. [17] demonstrated that phage MR-5 specific to *S. aureus* enhances the potential of phagocytic cells to kill intracellular bacteria.

In the course of bacterial infections, bacteriophages also interfere with eukaryotic cells, affecting inflammatory response mechanisms and innate immunity and even reducing transplant rejection [12]. Due to their protein structure, the presence of phages in the human or animal body can also directly or indirectly modulate immune processes. Phages can affect both innate immunity, through phagocytosis and cytokine reactions, and acquired immunity, by inducing antibody production and activating effector cells. They can therefore have a profound impact on the course of bacterial infections by modulating the immune response [18].

The microbiome of a healthy human being or animal, apart from very numerous bacteria, also contains a large number of bacterial viruses, i.e., bacteriophages, which form the phageome [19]. Some research [20] indicates that intestinal bacteriophages mainly show affinity for anaerobic bacteria and that as many as 75% to 99% of sequences from intestinal phages do not yet fully match any known viral genome. It has also been suggested that disturbing the homeostasis of the phageome can lead to intestinal dysbiosis, stimulating the development of type 1 diabetes and intensifying the development of inflammation in inflammatory bowel disease. However, the presence of phages can also cause changes in the characteristics of bacteria, especially in the case of formation of intracellular prophages. Examples include immunity to a superinfection, elimination of competition for bacterial colonization, the possibility of horizontal transfer of genes determining an increase in drug resistance in bacteria, and an increase in their virulence [16].

Bacteriophages also exhibit the ability to move from the intestinal environment to other systems and organs, such as the kidneys, liver, spleen, muscles, lymph nodes, and the circulatory system [19,21].

### 1.2. Transport of Phages Through Eukaryotic Cells

The affinity of bacteriophages for mucus activates their ability to penetrate cells and tissues, usually by endocytosis and transcytosis, e.g., in intestinal epithelial cells or through dendritic cells [18,22]. Phages can migrate through layers of epithelial cells and then enter the bloodstream and spread throughout the body, activating immune responses [18]. This is the result of interactions between phages and eukaryotic cell membranes via transmembrane mucins and specific and nonspecific receptors, which enable signal transmission in epithelial cells. The phenomenon of phage–epithelial transcytosis takes place in various types of epithelial cell layers (e.g., in the cells of the intestines, lungs, liver, kidneys, and brain) and is a response to the spread of phages in the body, during bacterial infection as well as in states of health [23,24].

**Figure 1:**
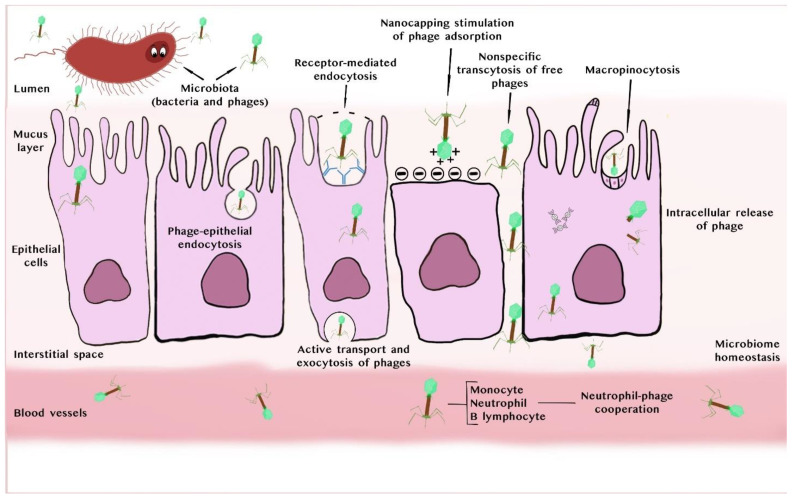
Schematic representation of various interactions and translocation of phages with epithelial cells adapted in part from [18,22,23,25] with our own modifications. Legend: Bacteriophages present on mucosal surfaces can be taken up by dendritic cells or epithelial cells by means of transcytosis or can diffuse through damaged barriers to reach internal tissues, including the bloodstream [18]. Contact between phages and eukaryotic cells is usually possible through extracellular factors such as glycoproteins and glycolipids forming a mucin layer which protects the cells. Adherence of phage particles to this layer creates an antimicrobial barrier, which reduces colonization by bacteria and epithelial cell death [18]. Beyond this layer, phages can be adsorbed (in just 30 s) directly to integrin and other T cell receptors (TCRs), or, alternatively, can bind directly to sialic acid residues [25]. Intensification of phage adsorption from the eukaryotic cell surface can be enhanced by modulating electrostatic interactions between the phage and eukaryotic cells using a nano-cap strategy, as demonstrated by [26]. Next, phage particles are taken up by epithelial cells and can then be degraded, leading to intracellular release of phage components and free phage DNA. Following endocytosis, phage nucleic acids can trigger TLR (Toll-like receptor) pathways, particularly those dependent on TLR9 receptors, thereby stimulating the acquired immune response [22]. Bacteriophages can also penetrate eukaryotic cells, which allows them to spread through the body, including the bloodstream. This is known as ‘phage transcytosis’. They can also move through the body through a ‘leaky gut’, which allows them to bypass the barrier of epithelial cells at damaged sites or sites of blood vessel perforation [18].

An example of bacteriophage interaction with eukaryotic cells and their translocation through them is illustrated in Figure 1.

The mechanisms by which bacteriophages activate B and T lymphocytes are not yet fully understood, as suggested by the latest research [12]. Earlier research, however [19], indicates that activation of B lymphocytes as a result of contact between dendritic cells and phages induces the production and release of anti-phage antibodies in the intestines and other organ systems. Bacteriophages have also been shown to influence the production of cytokines, such as IFN-γ and IL-6, due to activation of T cells in Peyer’s patches and mesenteric lymph nodes.

Bacteriophages are also currently believed to influence the immunity of eukaryotic organisms. In particular, phages present in the bloodstream have been shown to be able to modulate the innate and acquired immune response through contact with immune cells. Some studies have shown that despite the significant role of the intestine, it is not the only source of bacteriophages in the blood, and translocation of phages from the intestine to the bloodstream is irregular and weak [21]. Bacteriophages can penetrate the bloodstream at a rate of 98.5% via injection (i.v., i.m. s.c, or i.p.), 66.7% via inhalation, 50% by topical administration, and 41.1% through oral administration (oral cavity and gastrointestinal tract) [27].

There are two main aspects of interactions between phages and immune cells. The first is phage immunogenicity, i.e., the natural ability of phages to induce a specific immune response, especially the production of antibodies against phage antigens (mainly capsid proteins). The second aspect is the immunomodulatory activity of bacteriophages, i.e., their nonspecific effects on various functions of the major immune cell populations involved in both the innate and acquired immune response. In this case, bacteriophages can affect a variety of immune functions, such as phagocytosis or respiratory burst of phagocytic cells; antibody production; and proliferation of B cells, monocytes, and macrophages [28,29]. For example, it was suggested that bacteriophages may induce phagocytosis processes by adhesion and opsonization to bacterial cells, thereby increasing the ability of immune cells to recognize bacteria–phage complexes, which increases the phagocytic activity of neutrophils and macrophages [30,31].

Differences in the mechanisms of bacteriophage action on eukaryotic cells may also result from their modes of translocation. As shown by Sun et al. [32], bacteriophage translocation is related to their morphological structure, e.g., Caudovirales phages with large tails, the best-known representative of which is phage T4, use for this purpose a contractile envelope surrounding the tail tube, which contracts after detecting the host cell surface, causing puncture of the outer cell membrane. On the other hand, small, filamentous, tailless icosahedral bacteriophages, with the exception of microviruses, use channels encoded by host cells for translocation. Microviruses, a well-known representative of which is phage ΦX174, have quite specific conditions. In the studies of Jaźwiński et al. [33], it was suggested that specific phage H proteins may create a channel for DNA translocation through the cell wall. However, the authors indicated that this is only a hypothesis, and the exact mechanisms of translocation have not been identified.

The latest studies [34], which analyzed the translocation patterns of phages T4, ΦX174, and M13 across the intestinal barrier, showed that the highest level of association with eukaryotic cells and the highest translocation were exhibited by the M13 phage; in second place was phage T4, and the lowest translocation was observed in the case of phage ΦX174. However, no statistically significant differences were observed in the rate of translocation of the tested phages across the endothelial barrier [34].

The observed differences in the translocation of the tested phages resulting from their structure may affect the route of their translocation. Most phages with a filamentous structure, like M13, can migrate through mammary cells by macropinocytosis. In contrast, T4 and ΦX174 phages may use different endocytosis pathways. The least-understood mechanism is characteristic of microphages [35,36].

### 1.3. Anti-Inflammatory Effect of Bacteriophages

Bacteriophages can also exhibit anti-inflammatory activity, taking part in controlling inflammatory responses, e.g., in the following ways:

- Inducing an increase in the expression of the anti-inflammatory interleukin-1 receptor antagonist (IL-1RA);
- Stimulating the production and release of IL-10; blocking the expression of pro-inflammatory cytokines (IL-1α and -β and IL-6); and inhibiting the activity of Th1 cells, NK cells, and macrophages [1];
- Inhibiting the release of TNF-α (the main inflammatory cytokine), nuclear factor-κB (NF-κB), and C-X-C motif chemokine ligand 12a (CXCL12a);
- Inhibiting the activity of Toll-like receptor 4 (TLR4), whose activation stimulates the production and release of pro-inflammatory factors;
- Inhibiting the development of oxidative stress, e.g., by reducing overproduction of reactive oxygen species (ROS) in phagocytic cells—macrophages and neutrophils [12];
- Increasing the expression of anti-inflammatory factors, such as suppressors of cytokine signaling (SOCS3) or IL-1 (IL1RN) and IL-10 receptor antagonists [12];
- Inhibiting migration of neutrophils and granulocyte–macrophage colony-stimulating factor (GM-CSF) [37,38].

**Figure 2:**
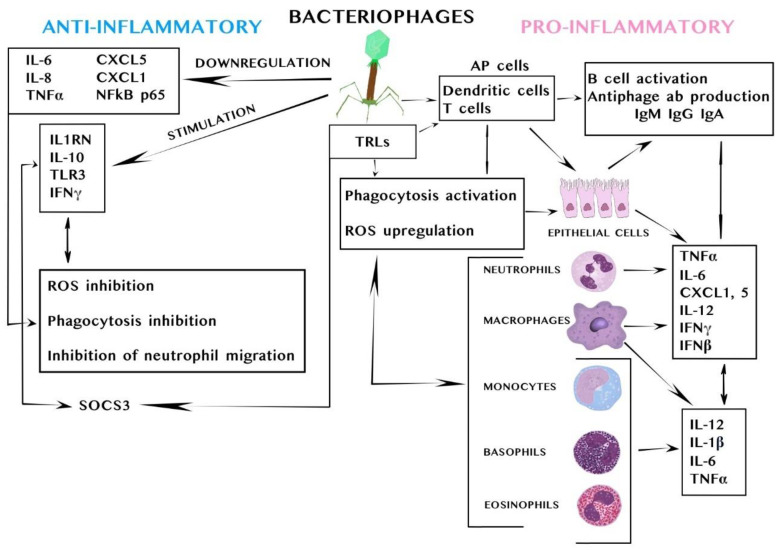
Pro- and anti-inflammatory mechanisms of action of bacteriophages on eukaryotic cells. **Legend:** Upon contact with immune cells, various pro- or anti-inflammatory cytokines are induced, which allows phages to influence the immune response. The interaction of bacteriophages and antigen-presenting cells (AP cells, e.g., dendritic cells) activates B lymphocytes to produce specific anti-phage antibodies. Antigen-specific T cells can then activate B cells to produce anti-phage antibodies. When cells involved in the immune response detect phages, they release cytokines, such as interferon (IFN)-β, interleukin (IL) 6, IL-10, and IL-12, which activate T and B cells. Phages can induce a cytokine response, but exactly which cells and pathways are responsible for these mechanisms is yet unknown [12]. Phages can also increase phagocytosis of bacteria by macrophages, especially as a result of opsonization of bacteria, which makes them more recognizable to the immune system [17]. Phages indirectly modulate phagocyte damage, which reduces ROS production by phagocytic cells, in part through absorption of bacteria by phage particles [39]. The mechanism reducing the destructive effect of ROS by eliminating bacterial infections is not yet fully understood [12]. The anti-inflammatory effect of phages may be associated with the direct interference of phage proteins in cell receptors, such as binding to cell integrins and TRL-like receptors, which activates cellular metabolism cascades, thus inhibiting the regulatory activity of integrin receptors present on cells involved in the host immune response and interfering with their natural inflammatory response [12].

Examples of the pro- and anti-inflammatory mechanisms of action of bacteriophages on eukaryotic cells are presented in Figure 2.

### 1.4. Examples of Experimental Studies with Bacteriophage-Caused Immunomodulatory Effects

The effect of bacteriophages on eukaryotic cells has been the subject of numerous studies. For example, a study by Przerwa et al. [39] showed that bacteriophage T4 inhibits phagocyte activity and reduces ROS production in response to infection with pathogenic *Escherichia coli* strains. This phenomenon seems to depend on specific phage–bacteria interactions, but, according to the authors, the precise mechanism is not fully understood.

A study on zebrafish (*Danio rerio*) exposed exclusively to bacteriophages specific to strains of *P. aeruginosa* showed that the anti-inflammatory mechanisms of bacteriophages are not dependent on their bactericidal activity. Moreover, the induction of an anti-inflammatory effect depends on the identification of phage capsid proteins through activation of the TLR receptor pathway [40,41]. An anti-inflammatory effect has been confirmed following injection of a cocktail of two phages of the family *Podoviridae* (GenBank vB_PaeP_PYO2, MF490236, and vB_PaeP_DEV, MF490238) and two of the family *Myoviridae* (vB_PaeM_E215, MF490241, and vB_PaeM_E217, MF490240), at titers of 5 × 10^8^ pfu/mL. Reduced expression of the IL-1-beta and TNF-alpha genes was observed in fish embryos, which translated into a significant reduction in neutrophil migration to the inflamed site [41]. The authors suggest that elucidating the regulatory mechanisms of bacteriophages will enable their use in preventing chronic inflammatory diseases.

Other authors [42] have shown that the properties of bacteriophages open the door to their application in diseases with symptoms of inflammation, such as *Clostridium* sp. infections and Crohn’s disease.

The anti-inflammatory properties of phages have been significant enough to reduce inflammation in lung and urinary tract infections in mice and even reduce the skin-graft rejection rate in mice [43].

In another study [44], the use of a phage cocktail containing phages specific to *Salmonella typhimurium* in drinking water for chicken broilers had a significant anti-inflammatory effect just one day after the birds had been infected with *S. typhimurium*. The anti-inflammatory effect and simultaneous antibacterial effect were manifested as inhibition of the increase in levels of pro-inflammatory cytokines, including IL-1β, IL-6, IFN-γ, IL-8, and IL-12, as well as stimulation of the production and release of anti-inflammatory cytokines (IL-10 and IL-4). Furthermore, the authors showed that the use of the experimental phage therapy in chickens had no adverse effect on the number and activity of lymphocyte subpopulations crucial to immune function. This suggests that phage therapy can be used in veterinary medicine without disturbing immune homeostasis, expressed as cytokine imbalance, abnormal percentages of key subpopulations of immune cells, and hyperactivity of the hypothalamic–pituitary–adrenal axis, which are common side effects of antibiotic treatment [44].

A recent study [45] carried out on seven-day-old chicks infected with *Salmonella enterica* showed that an antiviral response was initially induced in chickens that received 1 mL of a cocktail composed of two phages (vB_SenM-2 and vB_Sen-TO17) at a titer of 10^9^ pfu/mL per os for two weeks. However, the antiviral response was subsequently impaired via a blockade of one of the main pathways of innate antiviral immunity, i.e., cyclic GMP-AMP synthase (cGAS) and stimulator of interferon genes (STING), known as the cGAS–STING pathway, at the stage of phosphorylation of transcription factor IRF3. According to the authors, this reaction was caused by the inability of RNA polymerase III to recognize the phage DNA and produce dsRNA molecules crucial to stimulating the large protein complex necessary for phosphorylation of IRF3.

The number of phages adhering to the intestinal mucosa enables efficient selection of a specific microbiota, reducing the colonization capacity of pathogenic bacteria by limiting their adhesion to the mucosa [46]. The role of intestinal bacteriophages is believed to involve not only efficient regulation of the bacterial population colonizing the gut, but also regulation of local mucosal defense mechanisms (GALT and MALT) and enhancement of the activity of the probiotic microbiome [1].

The properties of bacteriophages open the door to their potential application in diseases in which intestinal inflammation plays a major role, such as infections caused by anaerobic bacteria of the genus *Clostridium* [1]. For instance, transfer of sterile fecal filtrate to the intestine or transplantation of purified fecal microbiota in people or mice can positively influence their health status by improving clinical parameters in *Clostridium* infections for up to seven months [47].

In another study [48], oral application of a cocktail of phages specific to *E. coli* strains K88, K99, and F41; *S. typhimurium*; *S. enteritidis*; and *C. perfringens* types A and C in weaned piglets resulted in anti-inflammatory and antioxidant effects by reducing the concentrations of IL-1β, IL-6, TNF-α, and myeloperoxidase. It is also worth noting that the treatment significantly reduced the concentrations of selected pathogens in the digestive tract and feces.

The pro-inflammatory effects of bacteriophages may be associated in part with stimulation of T cell proliferation, increased IL-6 production, and blast transformation of B lymphocytes and other cells involved in the immune response [49]. The pro-inflammatory effects of bacteriophages may depend on their individual properties, the route of administration, and the exposure time, as well as on the type of infection. For example, in the case of infections caused by Gram-negative bacteria, their breakdown results in an increase in the level of LPS—the main pro-inflammatory endotoxin. Higher doses of phages in treatment can also induce a proportional pro-inflammatory effect, as certain inflammatory cytokines have been detected only at high phage titers (10^9^ pfu/mL) [49]. The pro-inflammatory activity of bacteriophages stimulates the production of anti-phage antibodies, leading to the induction of the humoral immune response (Table 1).

Some bacteriophages used in therapy may exhibit both pro- and anti-inflammatory activity at the same time, as confirmed in research by Zhang et al. [62]. In that study, intramammary application of phages specific to *S. aureus* in cattle resulted in a reduction in the levels of cytokines TNF-α, IL-1β, IL-6, and IL-8 in mammary epithelial cells (MAC-Ts) stimulated with LPS, as well as a reduction in the levels of inflammatory mediators in the absence of LPS.

Apart from therapeutic uses of bacteriophages, owing to their immunomodulatory properties they can also be used in immunoprophylaxis. For instance, phage phi X174 specific to *E. coli* strains has been used for more than 30 years as an antigen to assess the humoral immune response in patients with primary and secondary immune deficiencies, such as severe combined immunodeficiency (SCID), X-linked agammaglobulinemia (XLA), X-linked hyper IgM syndrome, and Wiskott–Aldrich syndrome, as well as in bone-marrow recipients, people infected with HIV, and patients treated with immunosuppressants such as CTLA4-Ig [63]. Phage particles can also be used as carriers for vaccine antigens; however, the potential interactions between a given bacteriophage and immune cells must be investigated [64].

Apart from regulating the intestinal immune response against bacteria and viruses, bacteriophages can also play a role in the anti-tumor response. They have been shown to be capable of exerting a targeted effect on specific molecular determinants of cancer cells and inhibiting tumor growth by accumulating in cancer tissue. Some studies indicate that phages can be used as anti-cancer agents and carriers of imaging molecules and therapeutic agents [65].

For example, in the studies of Hajitou et al. [66], it was shown that the use of hybrid vectors containing fragments of a eukaryotic virus in combination with a bacteriophage as a representative of a prokaryotic virus in the form of a chimera (AAVP—adeno-associated virus/targeted M13 phage) was characterized by high efficiency in terms of affinity for various types of human malignant tumors, such as Kaposi’s sarcoma (KS1767), bladder cancer (UC3), prostate cancer (DU145), and mammary tumors (EF43-FGF4), induced in a mouse model. In order to confirm the obtained results, the authors used the cyclic ligand Arg-Gly-Asp (RGD-4C) located on the bacteriophage capsid, showing affinity for αv integrin receptors specifically expressed on the blood vessels of the tumors studied. The obtained results confirmed the possibility of delivering transgenic genes by the developed vectors only to the areas affected by the cancer process, thus sparing healthy organs, which is a very good prognostic factor in the diagnosis and treatment of cancer [67].

It should be emphasized that the forerunners of research using phage display were the Nobel Prize winners from 2018, Prof. Smith, G.P., and Prof. Winter, G.P., who developed the possibility of creating fusion proteins with antibodies or peptides [68,69]. The developed technique involves cloning the sequence of interest at the N- or C-terminus of the capsid protein, using the nonlytic filamentous phage M13. Currently, bacteriophage M13 is the most common and highly effective phage used in cancer therapies. Genetically modified bacteriophages used in these studies constitute a matrix (vector) for stable transport of genes, inducing cancer cell death [70].

A special role is ascribed to temperate phages, i.e., those that multiply through a lysogenic cycle. It is these bacteriophages that can be a source of transfer of genes involved in the metabolism of toxins, polysaccharides, and carbohydrates for bacteria, and in rare cases they can be a source of antibiotic resistance [28,43,71]. Some phages can modulate the antigenicity of bacteria by producing enzymes capable of modifying LPS O-antigens in microorganisms such as *Escherichia coli*, *Salmonella* sp., *Shigella* sp., and *Vibrio cholerae*, thus enhancing their virulence traits.

Another example of the effect of temperate phages on bacteria is the formation of prophages, which can directly affect the immune system of people and animals. For example, prophage SF370.1 encodes extracellular DNase Spd1, leading to DNA degradation in neutrophil extracellular traps (NETs), thereby increasing the invasiveness of M1 strains of *Streptococcus pyogenes* [72].

Bacteriophages can also be induced naturally from lysogenic bacteria. An elevated level of phage antibodies was observed more than 50 years ago in patients infected with *Staphylococcus* spp. between days 6 and 14 of the course of the disease [73]. It was suggested that this may be the effect of bacteriophage production by lysogenic *Staphylococcus* strains, leading to an increase in levels of anti-phage antibodies.

Previous research [4] has shown that anti-phage antibodies can be some of the most important factors limiting the therapeutic efficacy of phages. However, many recent studies evaluating the effect of phage antibodies indicate that the efficacy of experimental treatments can be varied.

‘Natural’ anti-phage antibodies have been shown to be present in sera from people and animals that have not been treated with bacteriophages, although the level of phage-neutralizing antibodies was low [28]. In another study, the presence of anti-phage antibodies against the most popular bacteriophage, T4, was confirmed in 80% of healthy human subjects who had never received phage therapy [74].

The presence of ‘natural’ anti-phage antibodies specific to *E. coli* and *M. haemolytica* bacteriophages was also observed in our own study [53] in calves which did not receive experimental phage preparations, in both prophylaxis and treatment (Table 2). It should be noted that the study concerned not only preparations containing whole phages but also selected phage proteins exhibiting immunogenic properties.

The presence of natural phage antibodies produced by immune cells in people and animals is ascribed to the ubiquitous occurrence of bacteriophages in the environment and constant contact with various phages. For example, bacteriophages have been detected in wastewater, water bodies, soil, food, and animal feed, as well as in the oral cavities (dental plaque and saliva) and gastrointestinal tracts of people and animals. They are also present in commercially available sera and vaccines [75,76].

The presence of anti-phage antibodies shown in our own research in sera from healthy calves which had had no physical contact with phage preparations indicates the presence of commensal bacteriophages in the natural microbiome (phageome) of the gastrointestinal and respiratory tracts of calves. This can be assumed to be due to the presence of saprophytic strains of *M. haemolytica* colonizing the nasal cavity and *E. coli* colonizing the gut, as part of the natural intestinal microbiome [53,77].

According to Archana et al. [54], the presence of natural anti-phage antibodies against *E. coli* indicates that bacterial strains making up the commensal microbiota in animals can also induce the development of other specific bacteriophages making up the intestinal phageome, which influences the production of phage-neutralizing antibodies. On the other hand, the absence of anti-phage antibodies for other microbes, such as *Klebsiella pneumoniae*, *Salmonella typhi*, *Pseudomonas aeruginosa*, and *Staphylococcus aureus*, may suggest that these bacteria should not be considered part of the commensal microbiota but pathogenic microbes [54].

The results of our own research confirm the suggestions of Nguyen et al. [23], who found that about 31 billion bacteriophage particles migrate into the human body every day via transcytosis through intestinal epithelial cells, translocation through a damaged epithelial barrier, or direct uptake from the intestinal lumen by intestinal dendritic cells, as well as cells of the lungs, liver, kidneys, and brain, influencing mechanisms of the host immune response [78].

It has also been demonstrated that commensal intestinal bacteria, including *E. coli*, containing prophage genetic material are capable of producing bacteriophages, which enables them to compete with other bacterial species colonizing the intestines [79]. This underscores the importance of studies showing that the intensity of the humoral anti-phage response can also vary depending on the route of administration of phage preparations. For example, Srivastava et al. [80] found that following intravenous administration of bacteriophage T7, despite stimulation of the humoral response, T lymphocytes were not involved in the cellular immune response. In a very early study in 1970 [57], intramuscular administration of a cocktail of bacteriophages specific to *Streptococcus lactis* strains AM_3_ and ML_1_ resulted in a negligible immune response, expressed as the IgG level, in both sera and milk whey. Somewhat higher immune parameters were observed following immunization of animals with a bacteriophage cocktail with Freund’s adjuvant. Following intramammary administration of a bacteriophage suspension, the authors demonstrated the induction of inflammatory reactions in the udder, resulting in changes in the structure and organoleptic properties of milk. For this reason, they did not recommend the administration of bacteriophages directly into the udder.

In our own research on calves, no statistically significant differences (*p* ≤ 0.05) were shown in the obtained results depending on the route of administration, i.e., nasal (*M. haemolytica* phages) or rectal (*E. coli* phages). The values were similar in both cases [53].

It has also been confirmed that irrespective of the route of administration (oral, nasal, or intraperitoneal) of bacteriophages to animals (mice, rats, and rabbits) and humans, they appear very rapidly in the bloodstream and in internal organs involved in immune processes (the spleen, liver, and thymus) [81,82]. The presence of bacteriophages is especially quickly confirmed in the blood, due to their ability to penetrate endothelial cell barriers. Rectal application in chinchillas of bacteriophages specific to *Pseudomonas aeruginosa*, *Salmonella enteritidis*, and *Escherichia coli* bacteria, in the form of suppositories, resulted in a significant concentration in the blood in just 30 min, while a similar concentration was noted in the urine within an hour [83]. Kawato and Nakai [82] confirmed the ability of some phages, particularly phage PPpW-4 against *Pseudomonas plecoglossicida* in goldfish, to penetrate the intestinal wall to the bloodstream within just 10 min after oral administration.

Some studies have shown that induction of an immune response also depends on the duration of treatment, the titer of phages, and their individual immunomodulatory properties [78]. For example, Łusiak-Szelachowska et al. [84] showed the strongest anti-phage activity (K > 18) following the topical application of a phage cocktail to the skin or mucous membranes and following simultaneous topical and oral administration. In addition, significant differences were confirmed in immunogenicity between bacteriophages, with the greatest activity noted for the phage specific to *S. aureus*, 676/Z.

Geller et al. [60], in a study on dairy cows, found that, following subcutaneous and intramuscular injection of bacteriophages specific to *Lactococcus lactis* strains, serum antibodies had a low neutralizing effect (k = 6–8) against small bacteriophages with isometric head structures. Much stronger bacteriophage neutralization was observed in the colostrum in comparison with sera. The titers in the colostrum were as much as 30 times as high as those obtained in the sera (Table 3).

Production of anti-phage antibodies can also be stimulated by some bacterial cell components present in phage preparations, such as lipopolysaccharides (LPSs). However, Łusiak-Szelachowska et al. [84] showed no negative effect of phage antibodies on the therapeutic or prophylactic effects of phages, and the levels of anti-phage antibodies varied. In several cases, the titers of antibodies against the phages studied were negligible, while in other patients the differences were statistically significant compared to the controls. According to the authors, the differences observed in antibody levels may be due to the varied immunogenicity of phages, even those infecting the same host, which may in part be explained by high variation in protein sequences in the structural proteins of phages.

Studies presented by many research centers indicate the significant role of bacteriophages present in the intestines of both humans and animals in protecting the host against numerous pathogens, including *E. coli*, *Salmonella enteritidis*, *Pseudomonas aeruginosa*, and *Clostridium* sp. [1].

Our own research [53] also showed that despite the presence of anti-phage antibodies in phage-treated calves, there was no negative effect on the experimental treatments using phage preparations specific to *E. coli* and *M. haemolytica* strains. The absence of a significant suppressive effect of antibodies induced by phages was confirmed in part by the lytic activity of bacteriophages remaining at a similar level, improved health parameters in the sick calves, and a protective effect lasting for about three weeks, i.e., the absence of cases of disease in calves.

Positive results of phage therapy have also been obtained in the treatment of udder diseases in dairy cattle, both clinical and subclinical. Application of a suspension of bacteriophages specific to various pathogens responsible for these infections not only did not cause a significant immune response but also reduced inflammation and clinical symptoms [85]. However, particular caution is required when considering the use of bacteriophages to prevent mastitis in dairy cattle. Application of a bacteriophage suspension to healthy udder quarters during lactation was shown to cause a significant increase in the somatic cell count in the milk from the quarters in which phages were applied. This also confirms that bacteriophages induce an immune response in udder tissue [86]. Data from in vivo studies and positive results of the application of bacteriophages to the udders of dairy cows are currently lacking.

## 2. Conclusions

The results of various experimental studies, including our own, demonstrate that specific anti-phage antibodies can be induced in humans and animals; however, in many cases, this has no significant effect on the efficacy of experimental phage treatments.

In order to prevent an overly intensive immune response against phages, it can be helpful to purify them to eliminate any unnecessary protein ballast and thereby limit the induction of an immune response that could disrupt the therapy process.

The immunomodulatory effect of bacteriophages on eukaryotic cells, especially in the course of infection, opens up promising prospects for the use of phages as natural tools in the treatment of bacterial, fungal, and viral diseases, especially immune diseases, particularly autoimmune diseases, including cancers.

The few published studies on the effects of bacteriophages on eukaryotic cells in the course of bacterial infections, particularly in farm animals and pets, show that there is an unquestionable need to continue this research.

## Figures and Tables

**Table 1:** Anti-phage antibodies in selected animal species following application of experimental phage formulations [50] with our own modifications.

Bacteriophage	Host Species	Administration Route	Identified Antibodies	Animal Species	Material Tested	References
GACP	*Enterococcus faecalis*	Intraperitoneal	IgG, IgM	Mice	Blood	[51]
φ26, φ27, φ29	*E. coli*	Rectal (suppositories)	IgG, IgA, IgM	Calves	Serum	[52]
Whole phages: Φ25, φA2, φA5	*M. haemolytica*	Nasal	IgG, IgA, IgM	Calves	Serum	[53]
Extracted proteins	*M. haemolytica*					
specific to *E. coli*	*E. coli*	Subcutaneous	Not identified	Rabbits	Blood	[54]
AbArmy ϕ1, AbNavy ϕ1, AbNavy ϕ2, AbNavy ϕ3, AbNavy ϕ4	*Acinetobacter baumannii*	Intraperitoneal	IgG2a, IgG2b	Mice	Serum	[55]
A3R, 676Z	*Staphylococcus aureus*	Per os	IgM, IgG	Mice	Plasma	[56]
Bacteriophages specific to *Streptococcus lactis*: AM3 and ML1	*Streptococcus lactis* AM 3 and ML 1 w	Subcutaneous	IgG	Cows	Serum Milk whey	[57]
		Intravenous		Rabbits	Serum	
Phages f1 and f2	*E. coli*	Intradermal	IgG	Sheep	Serum	[58]
Phages ΦX 174 and T 2	*E. coli*	Intracardiac	IgG	Piglets	Serum	[59]
C2, phages p335 and p013 on *L. lactis* ssp. *lactis* F7/2, phage kh on Lac	*L. lactis* spp. *lactis*	Subcutaneous injection in the neck and intramuscular injection in the rump	IgG	Cows	Colostrum Serum	[60]
Phage SE-W109	*Salmonella*	Subcutaneous	Polyclonal IgG	Rabbits	Serum	[61]

**Table 2:** Average concentrations of anti-phage antibodies, IgG, IgM, and IgA, in mg/L, in calf sera for bacteriophages specific to *E. coli* and *M. haemolytica* [53].

Estimated Parameters	FBS	Calves Untreated with Bacteriophages	Calves Treated with Bacteriophages
*E. coli* phages			
IgG	0.0032 ± 0.001	0.06 ± 0.009 *	0.74 ± 0.16 *
IgM	0.014 ± 0.008	0.096 ± 0.038 *	0.211 ± 0.052 *
IgA	0.001 ± 0.0008	0.02 ± 0.007 *	0.19 ± 0.09 *
M. haemolytica phages			
IgG	0.005 ± 0.0008	0.72 ± 0.017 *	0.95 ± 0.08 *
IgM	0.004 ± 0.0001	0.08 ± 0.03 *	0.99 ± 0.11 *
IgA	0.002 ± 0.0005	0.03 ± 0.001 *	0.23 ± 0.005 *
E. coli phage extracted proteins			
IgG	0.004 ± 0.0003	0.062 ± 0.001 *	0.83 ± 0.032 *
IgM	0.002 ± 0.0009	0.042 ± 0.002 *	0.21 ± 0.004 *
IgA	0.001 ± 0.0001	0.02 ± 0.008 *	0.201 ± 0.002 *
M. haemolytica phage extracted proteins			
IgG	0.007 ± 0.0003	0.071 ± 0.04 *	0.77 ± 0.13 *
IgM	0.004 ± 0.0002	0.05 ± 0.02 *	0.3 ± 0.04 *
IgA	0.001 ± 0.0001	0.03 ± 0.01 *	0.15 ± 0.03 *

Legend: FBS—fetal bovine serum; Ig—immunoglobulin; * significant differences (*p* ≤ 0.05).

**Table 3:** Examples of the effects of bacteriophage-neutralizing antibody titers obtained in various studies.

Type of Bacteriophage	Bacterial Host	Animal Species	Degree of Antibody Neutralization (K) or Serum Neutralization Titer (Dilution)	References
φ c2, ml3, sk1; φ p335, p013; φ kh, φ16–18	*Lactococcus lactis*	Cattle	3.8	[60]
φ25, φA2, φA5 φ26, φ27, φ29	*M. haemolytica* *E. coli*	Calves	5.053–5.57 6.74–7.28	[53]
Wild-type phage	*E. coli* *Klebsiella pneumoniae* *P. aeruginosa* *Salmonella typhi* *S. aureus*	Rabbits	1/10; 1/100 10x dilution >300 to complete neutralization at 28d10x, >300 to complete neutralization at 21d10x, >300 to complete neutralization at 28d	[54]
φX 174	*E. coli*	Piglets	1/5	[59]
Wild-type phage	*Streptococcus lactis*	Cows	1/400	[57]
		Rabbits	1/80	

Legend: nd—not detectable.
